# The complete mitochondrial genome of *Traulia minuta* Huang & Xia, 1985 (Orthoptera: Catantopinae)

**DOI:** 10.1080/23802359.2021.1915711

**Published:** 2021-07-05

**Authors:** Zhongying Qiu, Yuting Wang, Hao Yuan, Xuan Zhao, Ningyu Ru, Li Liu, Yuanyuan Cui, Yantong Liu

**Affiliations:** a School of Basic Medical Sciences & Shaanxi Key Laboratory of Brain Disorders, Xi’an Medical University, Xi’an, China; b School of Life Sciences, Shaanxi Normal University, Xi’an, China

**Keywords:** Mitochondrial genome, *Traulia minuta*, Catantopinae

## Abstract

In the present study, we obtained and annotated the complete mitochondrial genome (mitogenome) of *Traulia minuta*. The length of the whole mitogenome was 15,636 bp and the AT content of the complete mitogenome was 74.5%. All protein-coding genes (PCGs) started with typical ATN codon and ended with complete TAA/TAG codons except Nad5, which ended with incomplete T codon. The phylogenetic tree indicated that *T. minuta* was clustered together with *T. szetschuanensis*.

*Traulia minuta* Huang & Xia, 1985 is a grasshopper belonging to the *Traulia* Stål, 1873, Catantopinae, Acridoidae, Acridoidea, Orthoptera (Cigliano et al. 2020). The *Traulia* Stål, 1873, contained 54 species, distributed in Asia (Cigliano et al. 2020). Here, we obtained and annotated the whole complete mitochondrial genome (mitogenome) of *T. minuta*, with the accession number, MF113247. The sample of *T. minuta* was collected from Lai yang river (Si Mao, China) (101°19 N, 22°57 E) in 2007, and was deposited in Molecular and Evolutionary Lab in Shaanxi Normal University in China and the voucher number of the specimen was 20070728M1. The *T. minuta* mitogenome sequences were assembled with the Staden Package 1.7 (Staden et al. 2000). Geneious Prime (Kearse et al. 2012) and MITO (Bernt et al. 2013) were used for protein-coding genes (PCGs) annotation. tRNAscan-SE 2.0 (Lowe and Chan 2016), MITO (Bernt et al. 2013), and Geneious Prime (Kearse et al. 2012) were used for tRNA genes annotation. Two rRNAs and the non-coding region were confirmed by similarity blast with related species using Geneious Prime (Kearse et al. 2012).

The mitochondrial genome of *T. minuta* was a closed circular double-stranded structure with the length of 15,636 bp. It contained 13 PCGs (COX1-3, ND1-6, ND4L, Cytb, ATP6, and ATP8), 22 tRNAs, and two rRNAs (rrnS and rrnL) and a non-coding region. Among them, 14 genes (including four PCGs, eight tRNAs, and two rRNAs) are encoded in the N-strand, and the remaining 23 genes are encoded in the J-strand. AT content of the whole sequences was 74.5% (A 42.4%, T 32.1%, C 14.7%, and G 10.8%). The mitogenome had a compact structure with 11 overlaps, ranging from 1 to 8 bp in length. Two rRNA genes, rrnS and rrnL were 796 bp and 1317 bp in size, respectively. The non-coding region was 784 bp in length and the AT content was as high as 82.9%.

Thirteen PCGs were concatenated by SequenceMatrix v1.7.8 (Gaurav et al. 2011). We construct phylogenetic relationship based on 13 PCGs with related 15 species using the maximum-likelihood (ML) method by RAxML (Stamatakis 2006) with 1000 bootstrap replicated and GTR + G + R substitution model (Figure 1). The phylogenetic analysis showed that *T. minuta* was clustered together with *T. szetschuanensis*.

**Figure 1.**
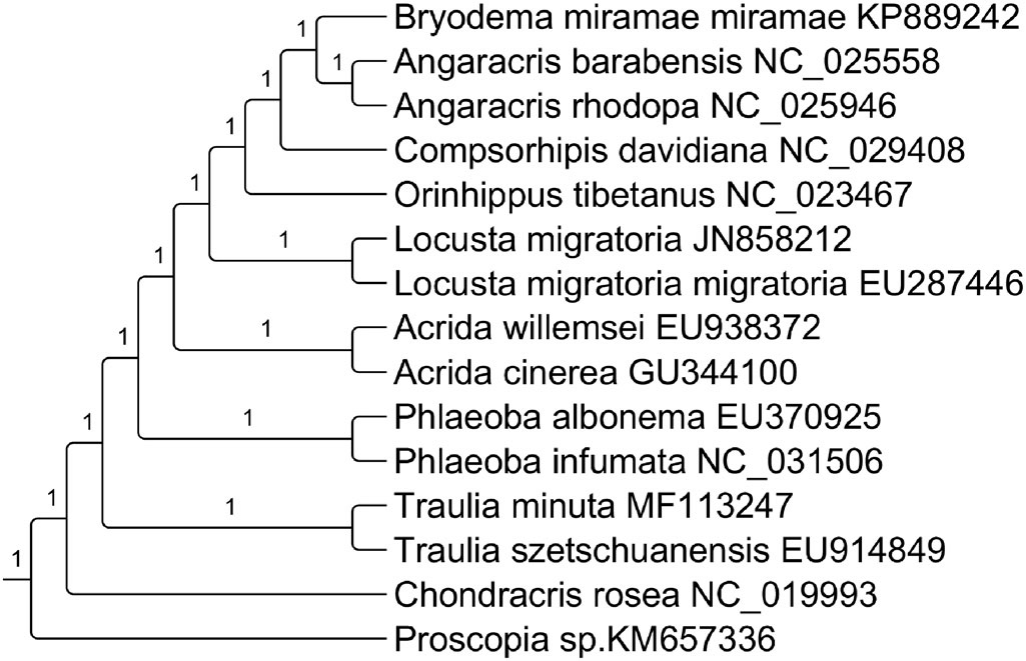
The maximum-likelihood tree inferred from 13 mitochondrial PCGs data. Proscopia sp. is used as the outgroup. The nodal numbers indicate the bootstrap values obtained with 1000 replicates. The species names and the Genebank accession number are shown on the right side of the tree.

## Data Availability

The data that support the findings of the present study are openly available in NCBI at https://www.ncbi.nlm.nih.gov/nuccore/MF113247, with the accession number, MF113247.
